# Isolation of a Nitromethane Anion in the Calix-Shaped Inorganic Cage

**DOI:** 10.3390/molecules25235670

**Published:** 2020-12-01

**Authors:** Yuji Kikukawa, Hiromasa Kitajima, Sho Kuwajima, Yoshihito Hayashi

**Affiliations:** 1 Department of Chemistry, Graduate School of Natural Science and Technology, Kanazawa University Kakuma, Kanazawa 920-1192, Japan; h.kitajima8923@stu.kanazawa-u.ac.jp (H.K.); skuwaji@stu.kanazawa-u.ac.jp (S.K.); 2 Japan Science and Technology Agency, Precursory Research for Embryonic Science and Technology, 4-1-8 Honcho, Kawaguchi 332-0012, Japan

**Keywords:** calixarene-like structure, polyoxometalates, nitronate, crystal structure, host–guest chemistry, anion receptor

## Abstract

A calix-shaped polyoxometalate, [V_12_O_32_]^4−^ (**V12**), stabilizes an anion moiety in its central cavity. This molecule-sized container has the potential to control the reactivity of an anion. The highly-reactive cyanate is smoothly trapped by **V12** to form [V_12_O_32_(CN)]^5−^. In the CH_3_NO_2_ solution, cyanate abstracts protons from CH_3_NO_2_, and the resultant CH_2_NO_2_^−^ is stabilized in **V12** to form [V_12_O_32_(CH_2_NO_2_)]^5−^ (**V12(CH_2_NO_2_)**). A crystallographic analysis revealed the double-bond characteristic short bond distance of 1.248 Å between the carbon and nitrogen atoms in the nitromethane anion in **V12**. ^1^H and ^13^C NMR studies showed that the nitromethane anion in **V12** must not be exchanged with the nitromethane solvent. Thus, the **V12** container restrains the reactivity of anionic species.

## 1. Introduction

Calixarene possesses a rigid conformation with a broader hydrophobic upper rim, a narrow hydrophilic lower rim, and a central annulus. Due to this attractive architecture, the host–guest chemistry of calixarene has been developed [1,2,3,4]. It can include various chemical moieties of neutral molecules, cations, and anions through hydrophobic, cation−π, anion−π, and hydrogen-bonding interactions [5]. The host property is finely tuned by the functional modification of the upper rim and/or the lower rim. Calixarene is utilized in various fields of science, such as catalysis, sensors, functional materials, analytical chemistry, electrochemistry, photochemistry, biochemistry, and pharmacy [6,7,8,9,10,11]. The cavity diameters, considering the van der Waals radii, around the upper rim of calix [4] arenes and calix [6] arenes are 3.8 Å and 5.0 Å, respectively [12].

**Figure 1:**
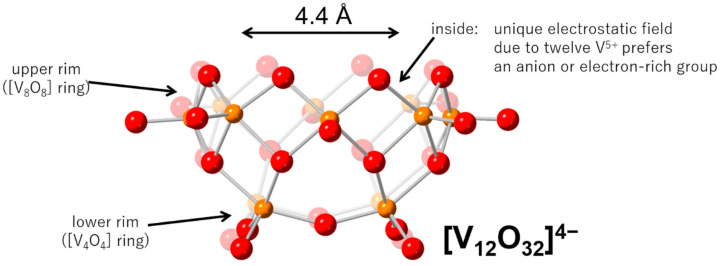
Schematic representation of a dodecavanadate. The red and orange spheres represent oxygen and vanadium atoms, respectively.

Polyoxometalates are a large family of metal-oxide cluster anions. They show several unique properties related to their well-defined structures [13,14,15]. Tungsten- and molybdenum-based polyoxometalates adopt lacunary structures by removing constituent elements. The lacunary type polyoxometalates act as inorganic multidentate ligands to stabilize several metal or metal-oxide cores. On the other hand, vanadium-based polyoxometalates formed by the condensation of VO_5_ square pyramids stabilize anionic moiety at the center of their clusters [16,17]. Up to now, various kinds of anions have been included in the vanadium-based polyoxometalates. Most anion-including polyoxometalates are sphere structures. [18] Although each electrophilicity directed to the base of the VO_5_ square pyramid is weak, the electrophilicity is strengthened by the condensation of the VO_5_ pyramids oriented to the center of a sphere to stabilize an anion. The distance between the central anion and its nearest neighbor vanadium is far from bonding distances, showing that the central anion is floating in the container. Among polyoxometalates composed of VO_5_ square pyramids, a ‘calix’-shaped dodecavanadate [V_12_O_32_]^4−^ (**V12**) is known [19,20,21,22,23,24,25,26,27,28,29,30]. The broader upper rim consists of eight edge-shared VO_5_ pyramids, with a 4.4 Å cavity entrance and a narrow lower rim consisting of four vertex-shared VO_5_ pyramids (Figure 1). This attractive structure was first reported by Day et al. in 1989 [19]. In the initial stage of the **V12** chemistry, the utility is limited to the guest exchange among nitriles because of the strong affinity between the nitrile group and the **V12** container [20,21,22]. Recently, we have developed the host–guest chemistry of **V12** [23,24,25,26,27,28,29]. By avoiding the usage of the nitrile group in the synthetic procedure by controlling the oxidation state of the vanadium sources, **V12** with guest-exchangeability to various electron-rich groups can be prepared [25]. In addition, the guest-removal from **V12** is successfully accomplished [28]. Importantly, during the guest removal, even in the solid state, one of the VO_5_ square pyramids of lower rim is flipped, and an oxygen atom is located at the center of the **V12** to fill the cavity. The structure is retrieved by the exposure to the guest vapor, such as acetonitrile, nitromethane, dichloromethane, 1,2-dichloroethane, bromomethane, CO_2_, or Br_2_. Thus, **V12** can be categorized as one of the guest-responsible Supramolecular Association–Dissociation Switches [3]. In the case of Br_2_, Br_2_ inserted into **V12** is polarized due to the unique charge distribution of the inside of **V12**, and the peak of the Br−Br vibration is detected in IR at 185 cm^−1^ [29]. Furthermore, the stabilization of several kinds of anions in **V12** has been reported [25,26,27]. The most interesting example is NO^−^-including **V12** [30]. While a standard chemistry text book introduces the electron structure of an anionic nitrogen monoxide, the isolation of anionic NO^−^ is very rare due to its stability and short life under normal experimental conditions.

Cyanide shows high nucleophilicity and acts as a ligand for several kinds of metal cations. The high nucleophilicity also makes it act as efficient Brønsted and Lewis base catalysts [31,32,33,34]. The discrete cyanide catalyzes the cyanosilylation of aldehydes, deprotonation, deacetylation, and Michael addition reactions. In this report, the host–guest chemistry of **V12** is applied to the reaction inhibitor. The addition of **V12** during the catalytic reaction with cyanide quenches the reaction to form cyanide-including **V12**. Successively, the guest exchange reaction proceeds to form nitromethane-anion-including **V12** ([V_12_O_32_(CH_3_NO_2_)]^4−^,**V12(CH_2_NO_2_)**) in nitromethane. The crystal structure and ^1^H and ^13^C NMR spectra are also discussed.

## 2. Results and Discussion

### 2.1. Reactivity of Cyanide and the Effect of the Addition of **V12**

**Figure 2:**
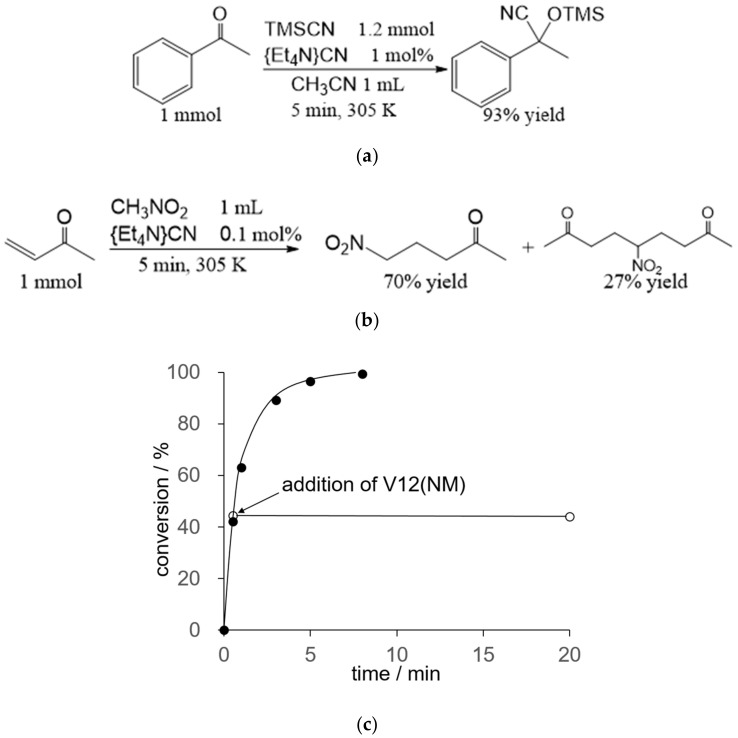
Catalytic performance of {Et_4_N}CN for (**a**) cyanosilylation, (**b**) Michael addition, and (**c**) time course profiles of Michael addition and the effect of the addition of **V12(NM)**. One equivalent of **V12(NM)** respective to {Et_4_N}CN was added 30 s after the reaction started.

Tetraethylammmonium cyanide {Et_4_N}CN is commercially available and stable. Bare cyanide in {Et_4_N}CN shows higher reactivity than that in metal cyanide complexes. In the presence of 1 mol% of {Et_4_N}CN, the reaction of acetophenone and trimethylsilyl cyanide gave the corresponding cyanohydrin trimethylsilyl ether with a 93% yield in 5 min (Figure 2). The turnover frequency reaches 19 min^−1^. This value is the highest level among the previous reports [35,36,37,38]. By the addition of **V12(NM)** into the reaction solution, **V12(NM)** was easily decomposed due to the formation of electrophilic trimethylsilyl cations and/or the successive formation of nucleophilic cyanide.

Cyanide also catalyzes Michael addition [34]. In the presence of 0.1 mol% of {Et_4_N}CN, the conversion of methyl vinyl ketone in nitromethane reached 97% in 5 min, to give 5-nitro-2-pentanone with a 70% yield and 5-nitro-2,8-nonanedione with a 27% yield (Figure 2). This reaction proceeded as follows. Nitromethane was deprotonated by the cyanide catalyst, and the reactive nitronate attacked the beta carbon to give the products. After 30 sec, about 40% of the methyl vinyl ketone was converted under the catalytic condition. By the addition of one equivalent of **V12(NM)** with respect to {Et_4_N}CN into the reaction solution after 30 sec, the reaction immediately stopped (Figure 2). Even if ten times the amounts of the catalyst and **V12(NM)** were used, the reaction did not proceed.

The ^51^V NMR of the reaction solution was measured. The results described below were beyond our imagination. From our previous report, it was assumed that the reaction was quenched by the incorporation of cyanide into the **V12**. However, this was not all. After 30 min from the addition of **V12(NM)** into the reaction solution, the ^51^V NMR showed t three signals at −564, −572, and −578 ppm, which are different signals from those of cyanido-including **V12** ([V_12_O_32_(CN)]^5−^, **V12(CN)**) (Figure S1). This spectrum is also different from that of **V12(NM)**. The spectrum most closely resembles that of acetate-including **V12** (([V_12_O_32_(CH_3_CO_2_)]^5−^, **V12(OAc)**), with three signals at −567 (sharp), −578 (broad), and −585 ppm (broad) (Figure S2).

The ^51^V NMR spectrum was monitored without the addition of methyl vinyl ketone (Figure S3). Compound **V12(NM)** in nitromethane showed three signals at −591, −596, and −606 ppm. Through the addition of {Et_4_N}CN, three signals at −578, −586, and −599 ppm due to **V12(CN)** were observed. With time, the intensity of the signals of **V12(CN)** decreased, and that of the three signals at −564, −572, and −578 ppm increased. The cold-spray ionized mass (CSI-MS) spectrum of the nitromethane solution of **V12(CN)** showed peaks at 1930 of {(Et_4_N)_6_[V_12_O_32_(CN)]}^+^ (Figure S4). With time, this peak intensity decreased and the intensity of the peak at 1964 assignable to {(Et_4_N)_6_[V_12_O_32_(CH_2_NO_2_)]}^+^ increased. Considering these results and the reaction mechanism, by the addition of **V12(NM)**, cyanide was actually trapped in **V12** to form **V12(CN)**; the successive deprotonation of nitromethane proceeded, and CH_2_NO_2_^−^ was stabilized in **V12** to form [V_12_O_32_(CH_2_NO_2_)]^5−^ (**V12(CH_2_NO_2_)**). Thus, the reaction of methyl vinyl ketone and CH_3_NO_2_ with {Et_4_N}CN catalyst stopped at the step of the formation of CH_2_NO_2_^−^ in **V12**.

### 2.2. Crystal Strucuture and Charactorization

**Figure 3:**
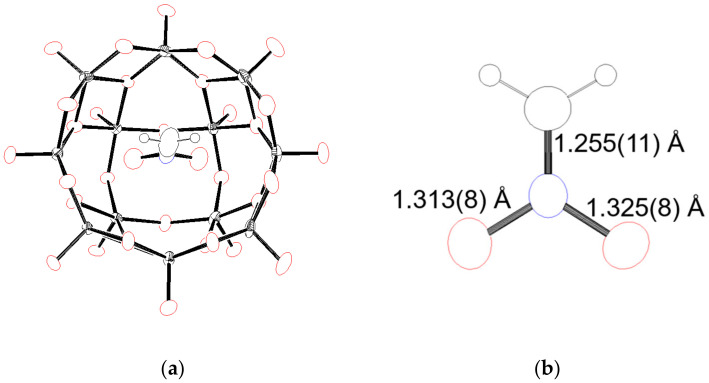
Ortep representations of (**a**) an anion section of **V12(CH_2_NO_2_)** and (**b**) a CH_2_NO_2_^−^ fragment in the cavity of **V12**. The black octant shading and spheres represent vanadium and hydrogen atoms, respectively. The red, blue, and black boundaries represent oxygen, nitrogen, and carbon atoms, respectively.

Fortunately, we can obtain crystals suitable for the X-ray structure analysis by the cation exchange from Et_4_N^+^ to Me_4_N^+^ (Table S1, Figure 3). The anion structure exhibits the typical **V12** structure with the guest moiety of CH_2_NO_2_^−^ in the concave. This is the first report on the crystal structure of nitromethane anions, as far as we know. Four CH_3_NO_2_ as crystalline solvents and five {Me_4_N}^+^ were determined, supporting the theory that the moiety in **V12** is a −1 charged anion. These results agreed well with the elemental and thermogravimetric analyses. In the case of neutral CH_3_NO_2_ as a guest, one of the oxygen atoms of the nitro group is inserted into the cavity, and the other oxygen is located at the same level of the entrance oxygen atoms of **V12** (Figure S5). On the other hand, two oxygen atoms of CH_2_NO_2_^−^ were packed into the concave of **V12**. The shortest distance between the nearest vanadium atom and an oxygen atom of CH_2_NO_2_^−^ is 2.538(6) Å, showing that CH_2_NO_2_^−^ is not directly bonded to vanadium centers. Although the visual aspect of **V12(CH_2_NO_2_)** is similar to that of **V12(OAc)**, each bond distance in the guest is different. CH_2_NO_2_^−^ possesses a 1.255(11) Å of C−N bond, and 1.325(8) Å and 1.313(8) Å of N−O bonds, while ^−^OAc possesses a 1.506(5) Å of C−C bond, and 1.257(4) Å and 1.260(4) Å of C−O bonds. Generally, a nitromethane anion exhibits resonance structures: anion charge locates on a carbon atom with a single C−N bond, and anion charge locates on oxygen atoms with a double C=N bond. The 1.255(11) Å bond distance of the C−N of CH_2_NO_2_^−^ shows the composition of a double bond between carbon and nitrogen atoms. During the above mentioned catalytic reaction, the formation of more reactive ^−^CH_2_−NO_2_ was restrained by the inclusion of CH_2_NO_2_^−^ in **V12**, and the reaction stopped.

The ^51^V NMR spectrum of **V12(CH_2_NO_2_)** in nitromethane is maintained for more than 60 min. In order to determine the ^13^C NMR for the nitromethane anion in **V12**, ^13^C-enriched nitromethane-anion-including **V12** was prepared. The ^51^VNMR spectrum showed isotope shift (Figure S1) [39]. The ^13^C NMR spectrum showed a peak at 109.3 ppm of nitromethane anions, in addition to peaks at 52.2 and 6.5 ppm of {Et_4_N}^+^. In an off resonance-decoupled ^13^C, a peak at 109.2 ppm was tripled, showing that two protons are attached to the carbon (Figure S6) [40,41]. The peak intensity of the nitromethane anion is maintained for more than 60 min, suggesting that the included ^13^C-nitromethane anion is not exchanged with the ^12^C-one derived from the nitromethane solvent. In the ^1^H NMR spectra in CD_3_NO_2_, a peak at 5.27 ppm due to the nitromethane anion was detected (Figure S7). The peak intensity is retained for more than 60 min, showing that the proton exchange reaction between the included nitromethane anion and nitromethane solvent does not proceed.

## 3. Materials and Methods

All of the reagents were obtained from commercial suppliers and were used without further purification unless otherwise noted. The solvents for the NMR measurements of the authentic **V12(CH_2_NO_2_)** were dehydrated by molecular sieve 4A. The **V12(NM)** and **V12(CN)** were synthesized following the literature [26].

For the synthesis of {Et_4_N}_5_[V_12_O_32_(CH_2_NO_2_)] and {Me_4_N}_5_[V_12_O_32_(CH_2_NO_2_)], the tetraethyl salt of **V12(CN)** (100 mg, 0.056 mmol) was dissolved in 5 mL of CH_3_NO_2_ and stirred for 1 h. The addition of an excess amount of diethyl ether with vigorous stirring gave a brown powder of {Et_4_N}_5_[V_12_O_32_(CH_2_NO_2_)]. The precipitates were collected by filtration and dried (85 mg, 83% yield). ^51^V NMR (in CD_3_NO_2_); *δ* = −564, −572, and −578 ppm. ^1^H NMR (in CD_3_NO_2_); *δ* = 5.28 (s), 3.40 (q), and 1.36 (t) ppm. ^13^C NMR (in CD_3_NO_2_); *δ* = 109.2, 52.2 and 6.5 ppm. The peak due to CH_2_NO_2_ was only observed by using a ^13^C-enriched carbon source. ^13^C-enriched [V_12_O_32_(^13^CH_2_NO_2_)]^5−^ was obtained by the dissolution of **V12(CN)** into ^13^CH_3_NO_2_. IR (Attenuated Total Reflection (ATR) without ATR correction): 2983, 2949, 2882, 1927, 1642, 1547, 1481, 1455, 1392, 1392, 1305, 1254, 1173, 1102, 1056, 1035, 973, 902, 853, 831, 750, 699, 628, 543, and 511 cm^−1^ (Figure S8). For the crystallographic analysis, tetramethylammonium salt was prepared. The tetraethylammonium salt of **V12(CH2NO2)** (91.7 mg, 0.05 mmol) was dissolved in 2 mL nitromethane. To this solution, 3 mL nitromethane solution of {Me_4_N}ClO_4_ (43.4 mg, 0.25 mmol) was added. The immediately-formed precipitates were removed by filtration and stood at 5 °C for 2 days. Elemental analysis calcd for {Me_4_N}_5_[V_12_O_32_(CH_2_NO_2_)]·4CH_3_NO_2_ (C_25_H_74_N_10_O_42_V_12_): C 16.70%, H 4.15%, N 7.79%; found: C 16.85%, H 4.17%, N 7.32%. The Thermogravimetric analysis data showed 14% mass-decreasing (the desorption of four CH_3_NO_2_ as crystalline solvent) by 180˚C.

The catalytic reaction was carried out as follows. Into a screw-capped test tube, 1 mmol substrate, 1 mL solvent, {Et_4_N}CN (catalyst) 1 mol% for cyanosililation and 0.1 mol% for Michael addition and naphthalene (internal standard) were added and stirred at 800 rpm at 305 K.

The IR spectra were measured with the ATR method (Zn/Se prism) on a JASCO FT/IR-4200 spectrometer. The ^1^H, ^13^C and ^51^V NMR spectra were recorded on a JEOL JNM-LA400. The thermogravimetry data were collected on a Rigaku Thermo plus EVO2 instrument with a temperature sweep rate of 10 °C/min under N_2_ flow (200 mL/min). The elemental analyses of C, H, and N were performed by the Research Institute for Instrumental Analysis at Kanazawa University. The GC analyses were performed on a Shimadzu GC-2014 with a flame ionization detector (FID) equipped with a InertCap Pure-WAX or ZB-WAXplus capillary column (internal diameter = 0.25 mm, length = 30 m).

The diffraction measurements of **V12(CH_2_NO_2_)** were performed on a Bruker D8 VENTURE diffractometer with graphite monochromated Cu Kα radiation (λ = 1.54178 Å). The data reduction and absorption correction were carried out using the APEX3 program [42]. The structural analyses were performed using APEX3 and WinGX [43]. The structures were refined by SHELXL-2013 [44]. The non-hydrogen atoms were refined anisotropically. The hydrogen atoms were positioned geometrically and refined using a riding model. The atoms in one of the tetramethylammoinium cations are restrained with a SIMU command. CCDC 2,041,581 contains the supplementary crystallographic data for this paper. These data can be obtained, free of charge, from The Cambridge Crystallographic Data Centre.

## 4. Conclusions

Calix-shaped dodecavanadate **V12** acts as an efficient trap for the reactive anion species. By dissolving cyanide-including **V12** in nitromethane, nitromethane is activated and the nitromethane anion—the reaction intermediate—is stabilized in **V12**, which enables the X-ray crystallographic analysis.

## Supplementary Materials

Figure S1: ^51^V NMR spectra of the reaction mixture of methyl vinyl ketone (1 mmol), CH_3_NO_2_ (1 mL), {Et_4_N}CN (10 μmol), and **V12(NM)** (10 μmol) after 20 min., Figure S2: ^51^V NMR spectra of (**a**) [V_12_O_32_(^13^CH_2_NO_2_)]^5−^, (**b**) **V12(CH_2_NO_2_)**, (**c**) **V12(OAc)**, (**d**) **V12(NM)** and (**e**) **V12(CN)** in nitromethane, Figure S3: ^51^V NMR spectra of the dehydrated-nitromethane solution of (**a**) **V12(NM)**, and {Et_4_N}CN (10 μmol), and **V12(NM)** (10 μmol) after (**b**) 1 min, (**c**) 5 min, (**d**) 10 min and (**e**) 20 min, Figure S4: CSI-MS spectra (positive mode) of the nitromethane solution of **V12(CN)** (**a**) just after dissolution, (**b**) after 10 min and (**c**) after 30 min, Figure S5: Anion structures of (**a**) **V12(CH_2_NO_2_)**, (**b**) **V12(OAc)**, (**c**) **V12(NM)** and (**d**) **V12(CN)**, Figure S6: (**a**) Decoupling and (**b**) off resonance-decoupled ^13^C NMR spectra of **V12(CH_2_NO_2_)** in CD_3_NO_2_, Figure S7: ^1^H NMR spectrum of **V12(CH_2_NO_2_)** in CD_3_NO_2_, Figure S8: IR spectrum of **V12(CH_2_NO_2_)** (ATR without ATR correction), Table S1: Crystallographic data for **V12(CH_2_NO_2_)**.
