# Supplementary material for: Isolation of a Nitromethane Anion in the Calix-Shaped Inorganic Cage
Source: Molecules. 2020 Dec 1;25(23):5670. doi: 10.3390/molecules25235670 (PMC7729913; doi:10.3390/molecules25235670)
Supplement: Supplementary file 1 [file molecules-25-05670-s001.pdf]

Supplementary Materials

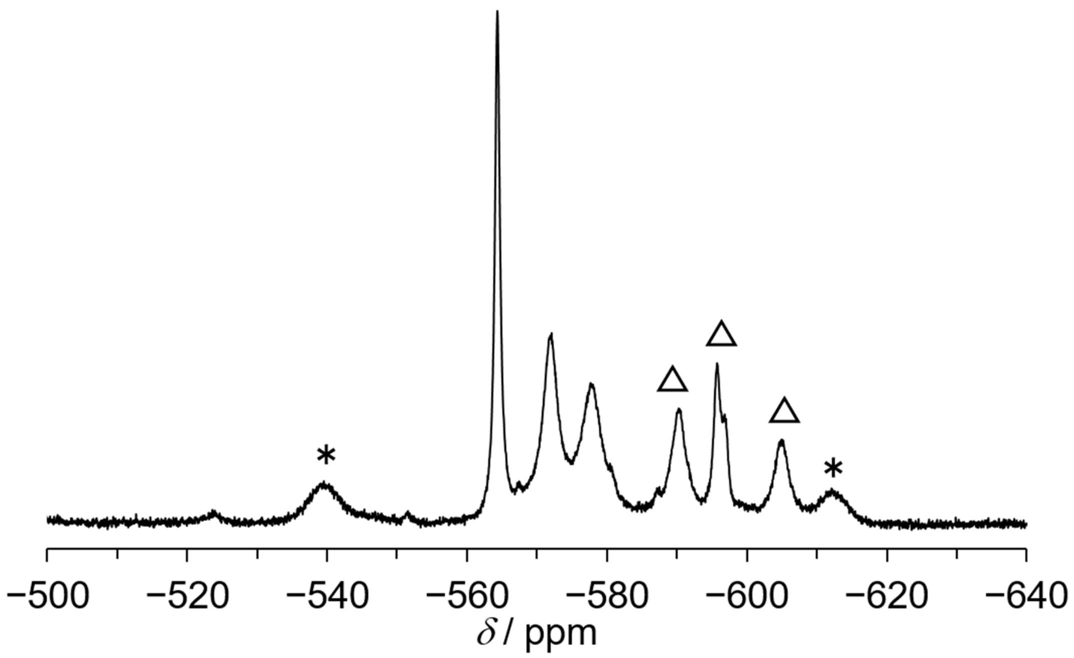

Figure S1. ^{51}V NMR spectra of the reaction mixture of methyl vinyl ketone (1 mmol), CH_3NO_2 (1 mL), $\{Et_4N\}CN$ (10 μmol), and **V12(NM)** (10 μmol) after 20 min. The asterisks and triangles represent the peaks due to the hydrolysis products ($[V_5O_{14}]^{3-}$) and **V12(NM)**, respectively.

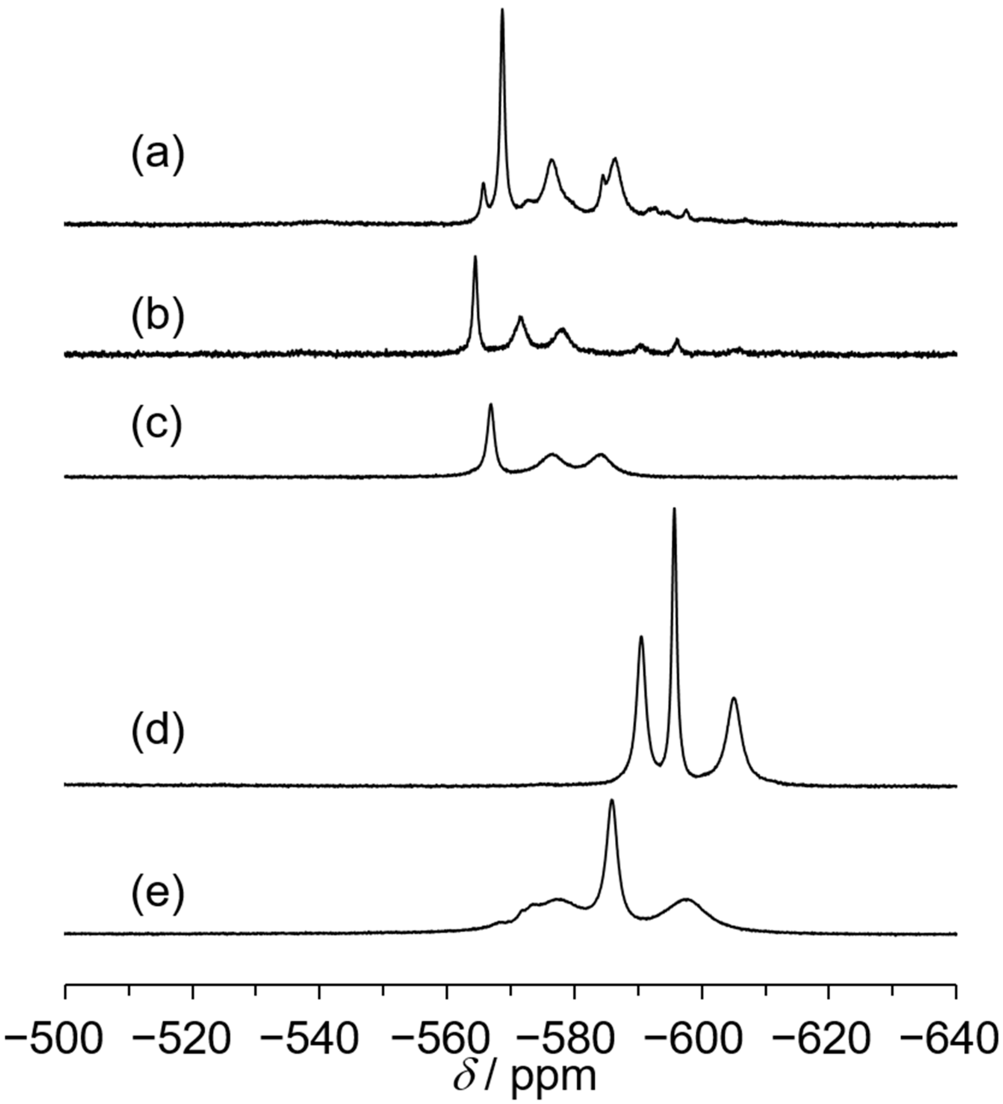

Figure S2. ^{51}V NMR spectra of (**a**) $[V_{12}O_{32}(^{13}CH_2NO_2)]^{5-}$, (**b**) **V12(CH$_2NO_2$)**, (**c**) **V12(OAc)**, (**d**) **V12(NM)** and (**e**) **V12(CN)** in nitromethane. The isotope shift was observed in spectrum (**a**) [39].

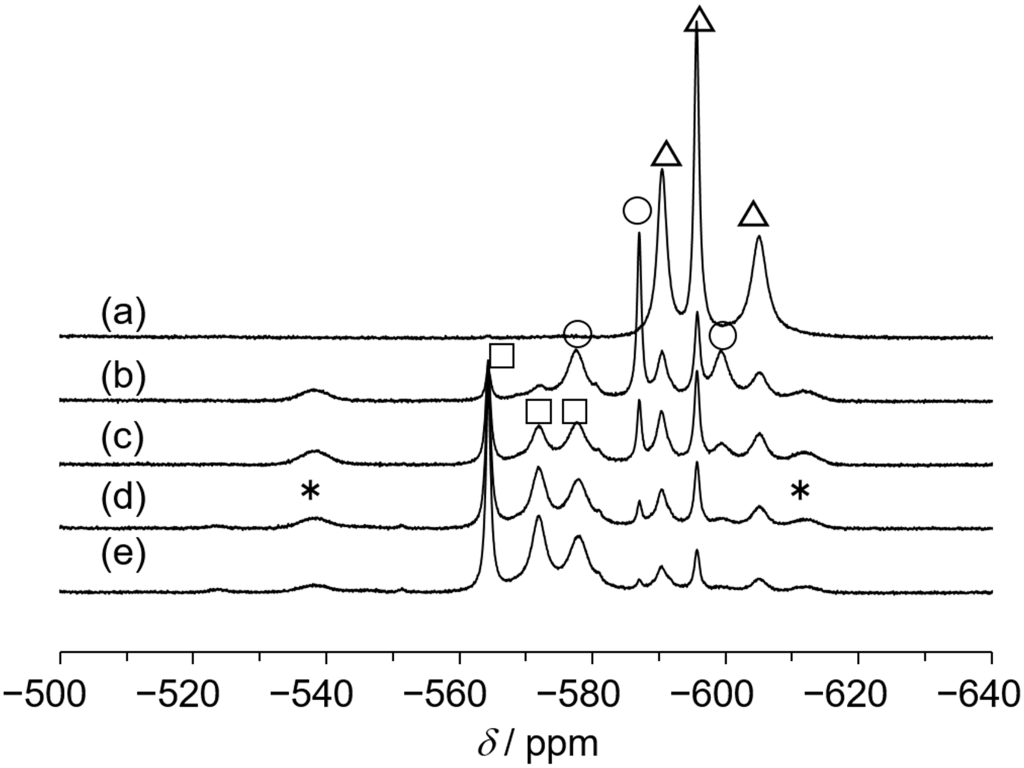

Figure S3. ^{51}V NMR spectra of the dehydrated-nitromethane solution of (**a**) **V12(NM)**, and {Et_4N}CN (10 μmol), and **V12(NM)** (10 μmol) after (**b**) 1 min, (**c**) 5 min, (**d**) 10 min and (**e**) 20 min. The asterisks, circles, triangle and square represent the peaks due to the hydrolysis products ($[V_5O_{14}]^{3-}$), **V12(CN)**, **V12(NM)**, and **V12(CH_2NO_2)**, respectively.

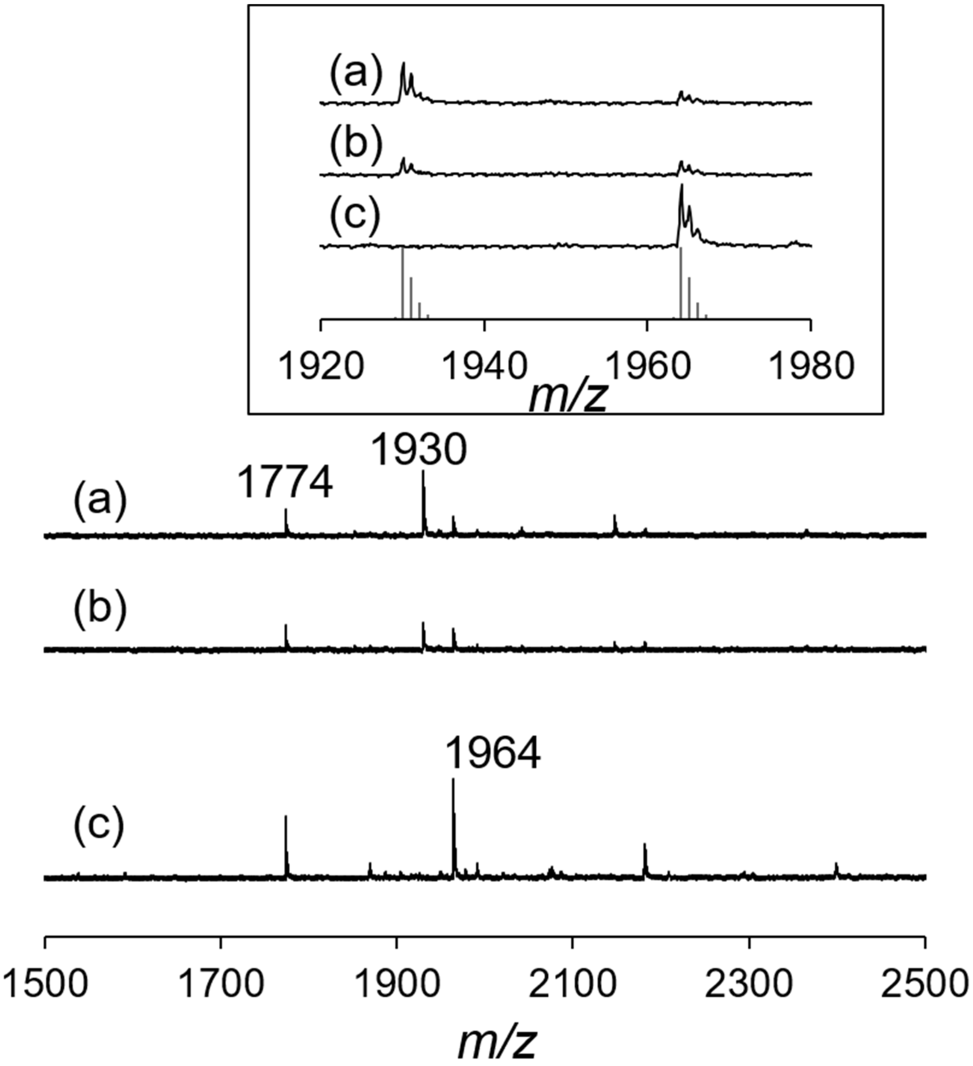

Figure S4. CSI-MS spectra (positive mode) of the nitromethane solution of **V12(CN)** (**a**) just after dissolution, (**b**) after 10 min and (**c**) after 30 min. The peak at *m*/*z* = 1774 is due to $\{(Et_4N)_5[V_{12}O_{32}]\}^+$. Neutral guest moiety was not detected even in the case of $[V_{12}O_{32}(CH_3CN)]^{4-}$, which shows strong affinity between a guest molecule and a **V12** host. The lines in the insertion represent the distribution of $\{(Et_4N)_6[V_{12}O_{32}(CN)]\}^+$ at *m*/*z* = 1930 and $\{(Et_4N)_6[V_{12}O_{32}(CH_2NO_2)]\}^+$ at *m*/*z* = 1964.

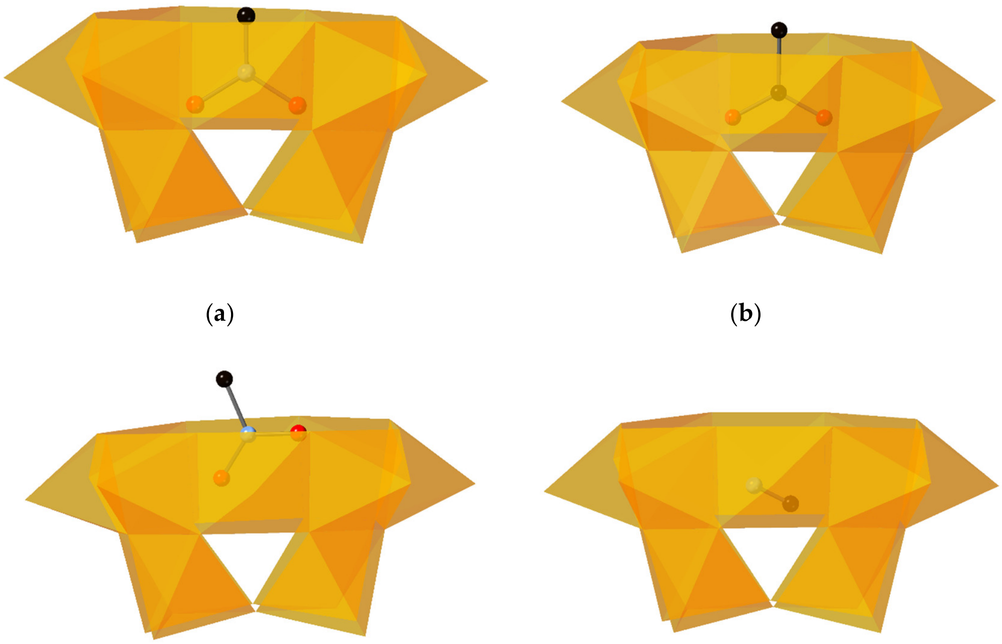

(**a**) (**b**)

(**c**) (**d**)

Figure S5. Anion structures of (**a**) **V12(CH_2NO_2)**, (**b**) **V12(OAc)**, (**c**) **V12(NM)** and (**d**) **V12(CN)**. The orange square pyramids represent the VO_5 unit. The red, blue, and black spheres represent oxygen, nitrogen, carbon atoms, respectively. The hydrogen atoms are omitted for clarity.

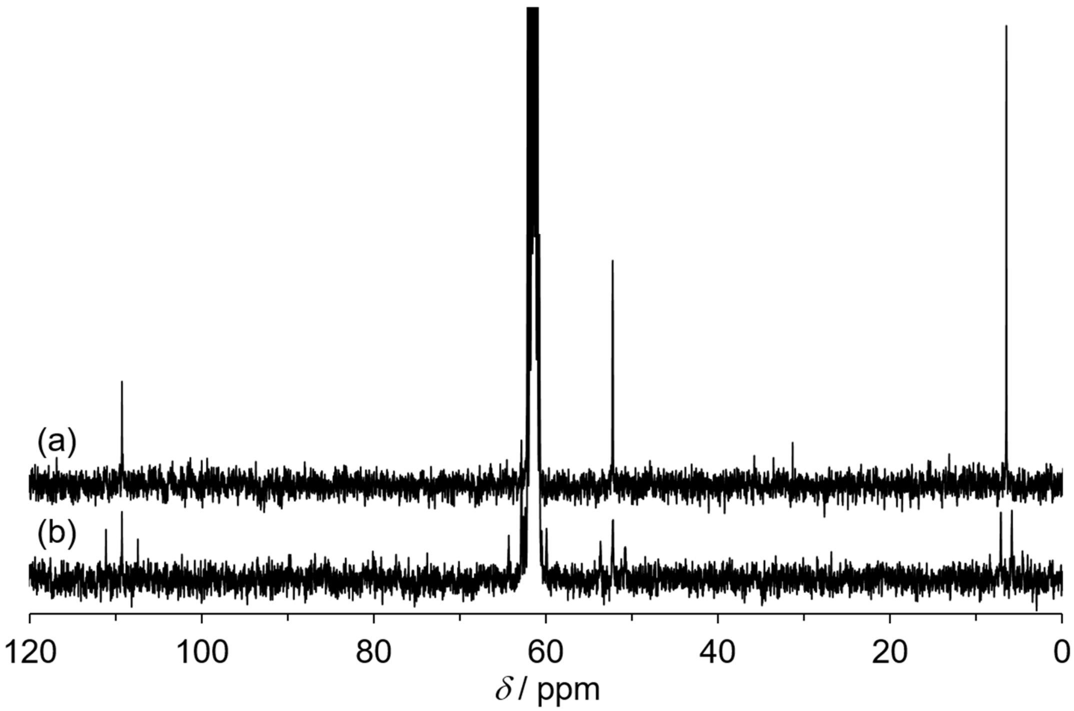

Figure S6. (**a**) Decoupling and (**b**) off resonance-decoupled ^{13}C NMR spectra of **V12(CH_2NO_2)** in CD_3NO_2.

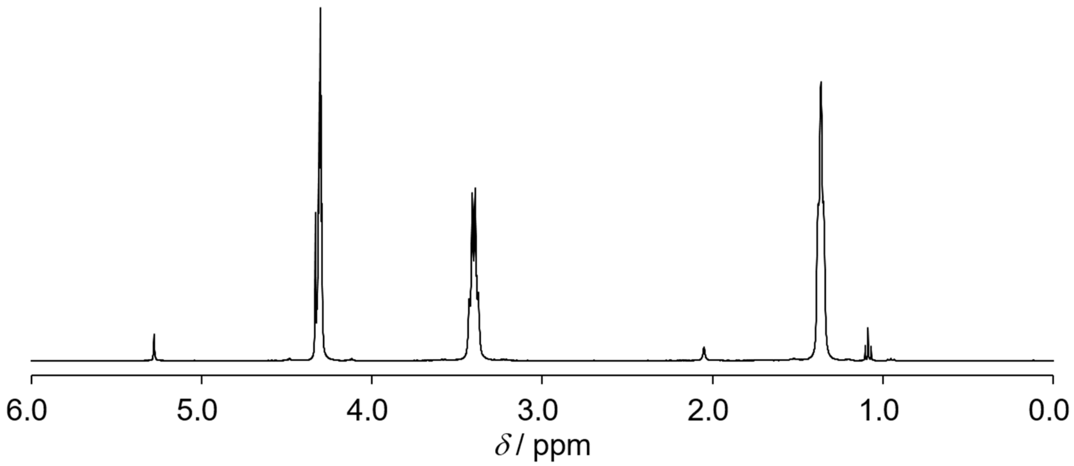

Figure S7. ^{1}H NMR spectrum of **V12(CH$_2$NO$_2$)** in CD_3NO_2.

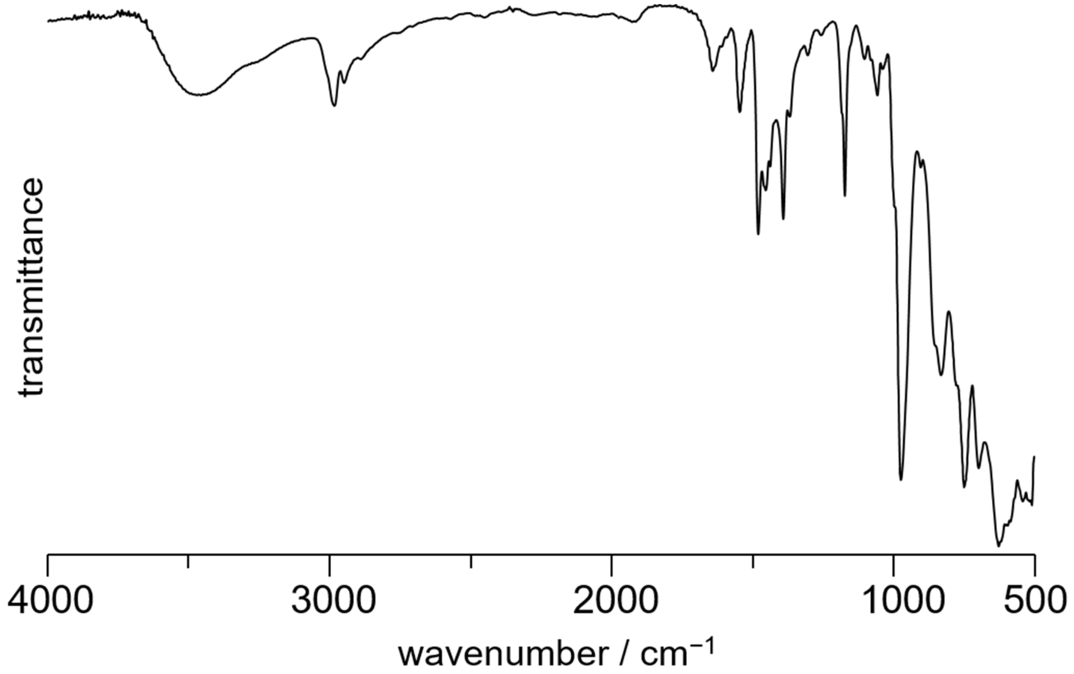

Figure S8. IR spectrum of **V12(CH_2NO_2)** (ATR without ATR correction).

Table S1. Crystallographic data for **V12(CH_2NO_2)**.

**Formula**	**$C_{25}H_{74}N_{10}O_{42}V_{12}$ ($\{C_4H_{12}N\}_5[V_{12}O_{32}(CH_2NO_2)]\cdot 4CH_3NO_2$)**
formula weight	1798.22
absorption correction type	Multi-scan
crystal system	triclinic
space group	*P*–1 (#2)
a (Å)	11.8658(5)
b (Å)	13.4272(6)
c (Å)	20.5836(9)
α (deg)	97.4890(10)
β (deg)	98.388(2)
γ (deg)	92.6090(10)
V (Å^3)	3209.7(2)
Z	2
μ (mm^{-1})	14.879
number of reflections	12137
number of parameters	782
number of restraints	24
data completeness	0.945
highest difference peak	1.767
deepest hole	−1.653
R_1 ($I > 2\sigma(I)$)	0.0719 (for 11132 data)
wR_2	0.1996 (for 12137 data)
